# Establishing content-validity of a disease-specific health-related quality of life instrument for patients with chronic hypersensitivity pneumonitis

**DOI:** 10.1186/s41687-020-00282-x

**Published:** 2021-01-14

**Authors:** Kerri I. Aronson, Maha Ali, Evgeniya Reshetynak, Robert J. Kaner, Fernando J. Martinez, Monika M. Safford, Laura C. Pinheiro

**Affiliations:** 1 Division of Pulmonary and Critical Care Medicine, Department of Medicine, Weill Cornell Medicine New York, New York, USA; 2 Division of General Internal Medicine, Department of Medicine, Weill Cornell Medicine, New York, NY USA; 3 Department of Genetic Medicine, Weill Cornell Medicine New York, New York, USA

**Keywords:** Cognitive interviewing, Chronic hypersensitivity pneumonitis, Health-related quality of life

## Abstract

**Background:**

Chronic Hypersensitivity Pneumonitis (CHP) is caused by an immune mediated response in the lung tissue after exposure to an inhaled environmental antigenic stimulant. We previously documented the ways in which CHP impacts patients’ lives and have now developed a disease-specific instrument, the CHP-HRQOL instrument, to measure health-related quality of life (HRQOL). The objective of this study was to assess content validity for the CHP-HRQOL.

**Methods:**

Cognitive interviews were conducted among adults with CHP. The instrument was revised and refined between each round of interviews. Feedback was obtained on the instructions, items, response options, and recall period. Items where participants had difficulty with comprehension, wording, or misinterpretation were marked by the interviewer and participant feedback was reviewed to make revisions, add or delete items when appropriate. Readability statistics were calculated using Flesch-Kincaid grade level and reading ease scores.

**Results:**

Ten participants were interviewed over three rounds, with revisions made to the questionnaire in an iterative process. In the initial 39 item instrument, we identified 7 items where two or more participants reported difficulty. Participants preferred a four-week recall period (compared to a two-week recall period) and response options with a 5-point response scale. The final version of the CHP-HRQOL includes 40 items with a median reading level between 6th and 7th grade.

**Conclusion:**

The CHP-HRQOL instrument demonstrated high content validity and is ready for psychometric testing in further validation studies.

**Supplementary Information:**

The online version contains supplementary material available at 10.1186/s41687-020-00282-x.

## Plain English summary

Chronic Hypersensitivity Pneumonitis (CHP) is a lung disease caused by an inhalational exposure in the environment to an organic antigen such as mold or bird feathers. Patients with CHP have variable clinical courses and the tests we currently use to measure disease status, such as pulmonary function testing, are not the outcomes that are truly important to patients. The impact that CHP has on patients’ quality of life can be profound. As more CHP patients are enrolled in therapeutic trials, there is a need for a patient-centered outcome specific to CHP patients. Our prior research with CHP patients identified components of quality of life that are not captured completely in currently available surveys. In response to this deficiency, we developed a disease-specific health related quality of life survey for CHP patients. The purpose of this study is to document “content validity” of the survey or the ability of the instrument to measure the important concepts specific to the CHP population that it was designed to measure. By documenting content validity, we also ensure that the questions, instructions, and answer choices are comprehensible, and the wording is appropriate. We performed in-depth interviews with patients asking for their feedback on the survey instructions, questions, and answer choices. We used this feedback to revise and refine the survey. Based upon the results of the survey revisions and interviews we have documented that the survey is understandable and appropriate for CHP patients and is ready for the next stages of survey validation.

## Introduction

Chronic Hypersensitivity Pneumonitis (CHP) is a subset of interstitial lung disease (ILD) that is caused by inhalation of an environmental antigen which results in inflammation and fibrosis of the lung tissue. The estimated annual prevalence of Hypersensitivity Pneumonitis in the United States is 1.67–2.71 cases per 100,000 persons and 50–63% of those are classified as chronic (CHP) [1]. The clinical course of the disease is heterogeneous and outcomes are highly variable from patient-to-patient [2]. In most therapeutic studies with ILD patients, it is common practice to report outcomes such as lung function testing [3–5]. We are aware, however, that reporting of such clinical objective measures does not adequately represent outcomes that are of interest to ILD patients [6]. Health-Related Quality of Life (HRQOL) is increasingly recognized as a valuable endpoint for patients living with ILD, and is now included more often in clinical trials and observational studies [7–13].

In addition to the common pulmonary symptoms of cough and shortness of breath, patients with CHP have poor HRQOL as compared to other ILDs when measured by generic HRQOL measures such as the 36 item short form survey (SF-36) [14]. In order to uncover the determinants of HRQOL in patients with CHP, we recently completed a qualitative research study utilizing semi-structured interviews with CHP patients to better understand patient experiences, perceptions, and expectations of living with CHP. From these data, we developed a thematic framework that incorporated several key themes including: 1. suffering due to lack of knowledge and uncertainty, 2. hypervigilance, 3. self-perception and identity, 4. psychosocial impacts, 5. physical impacts, and 6. interpersonal impacts. Each key them included several subdomains. The ability to identify and avoid the antigen responsible for the lung disease played a prominent role in all themes, and is a unique factor in CHP as contrasted to other ILDs. A detailed description of the framework is provided in our previously published study [15]. To date, existing HRQOL generic and pulmonary disease-specific reported outcome (PRO) instruments that have been used in ILD research do not comprehensively cover these CHP-specific quality of life themes [11, 16–19]. For example, the King’s Brief Interstitial Lung Disease (KBILD) survey does not include items related to knowledge, self-perception and identity, medication side effects, impacts on family and interpersonal interactions, environmental exposures, or fatigue, all of which we found to be important to patients living with CHP .

We are now at the cusp of an ever-expanding field of CHP research. This includes the establishment of diagnostic criteria [20, 21], efforts to improve methods for antigen detection and avoidance, and therapeutic clinical trials [2, 3, 22, 23]. As such, there is a need for a reliable and valid HRQOL measure for use in CHP. Due to the lack of a comprehensive instrument, we sought to develop a disease-specific HRQOL instrument for patients with CHP for use in both research and clinical settings. The overall objective of this study was to determine the content validity of the instrument, a crucial step in the initial stages of survey development and valildation [24]. Here we describe, in detail, the cognitive interview process utilized to document content validity of the disease-specific CHP-HRQOL instrument [25–27].

## Methods

### Participants and setting

Participants were recruited from the Pulmonary outpatient practice at Weill Cornell Medicine (WCM), a quaternary referral center. Adults older than 18 years with a diagnosis of CHP as designated by multidisciplinary discussion were recruited for the study. Eligible participants were fluent in the English language and willing to undergo an audiotaping of the cognitive interview. Individuals who were unable to read the questionnaire due to cognitive impairment were excluded from the study. We used purposive sampling to ensure that there was a heterogeneous sample of participants specifically with regards to gender, the duration of disease, severity of disease (as reflected by pulmonary function test (PFT) values and the need for supplemental oxygen), and the ability to identify the antigen source [28]. The WCM Institutional Review Board approved the study (Protocol #1802018963). Participants read and signed informed consent prior to participation in the study.

### Survey instrument

Prior to item generation, the standard of practice according to the Food and Drug Administration (FDA) and the International Society for Pharmacoeconomics and Outcomes Research (ISPOR) is to conduct qualitative research for concept elicitation to develop a thematic framework [25, 29]. We previously performed a qualitative research study for concept elicitation and developed a CHP quality of life thematic framework [15]. This framework informed the content and wording for the items in the instrument. All of the six key CHP-quality of life themes (previously described in the introduction) were represented in the instrument and the subdomains of each theme were represented by specific items in the instrument. An initial instrument comprised of 39 items was drafted. Each theme was represented by the following number of questions: 1. suffering due to lack of knowledge and uncertainty, 2 questions, 2. hypervigilance, 4 questions, 3. self-perception and identity, 5 questions, 4. psychosocial impacts, 11 questions, 5. physical impacts, 8 questions, and 6. interpersonal impacts, 7 questions. Response options were chosen for groups of items using polyomotous response scales with likert-type items of 7 for some groups and 5 for others. Recall period was discussed extensively amongst the research team and initially set at 2 weeks based upon several criteria including: clinician input and experience in caring for CHP patients, chronic features of the disease, and references to other ILD HRQOL PRO measures [19].

### Cognitive interviewing process

**Fig. 1:**
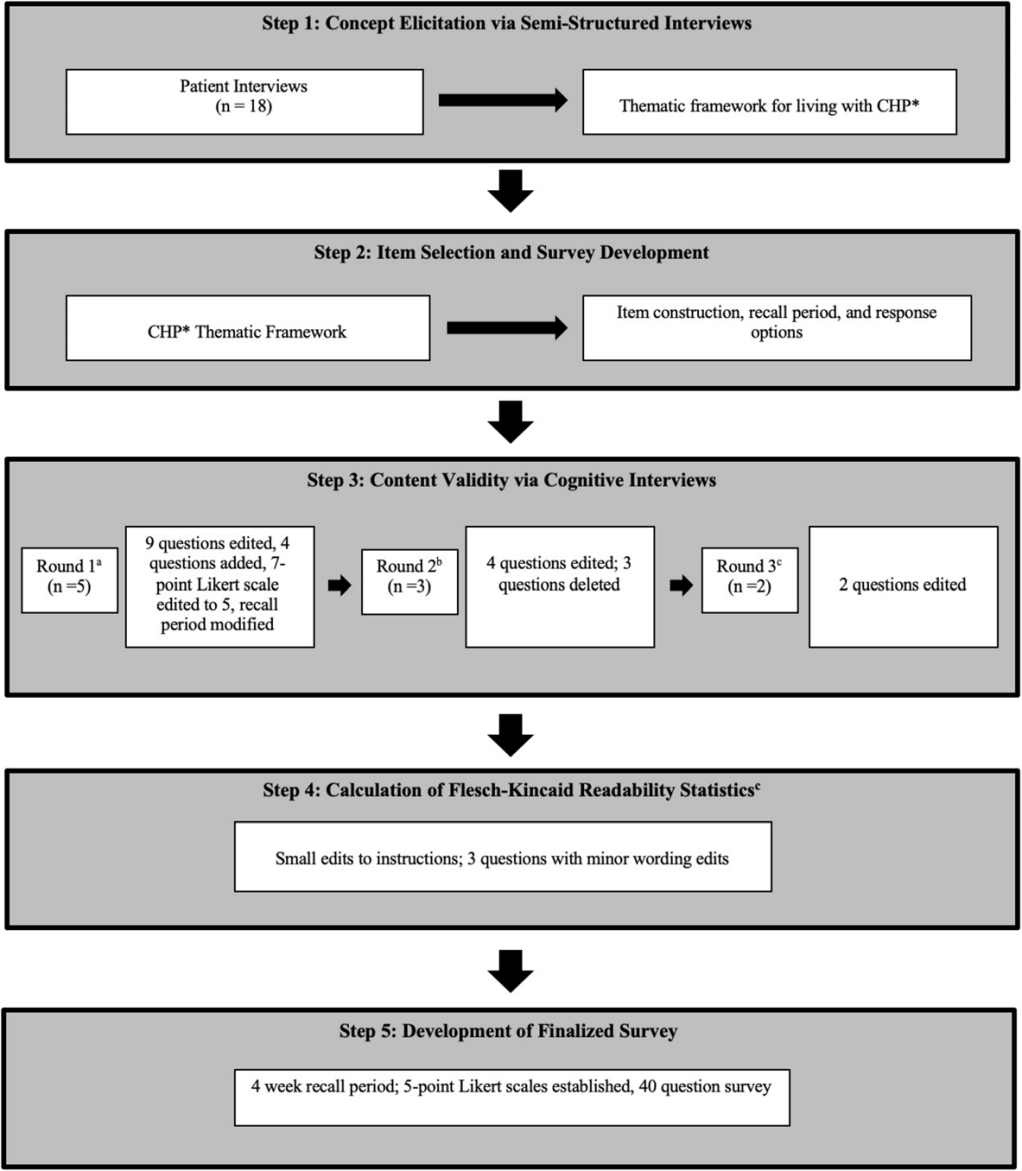
Methodology of Survey Development Using Cognitive Interviews. Key--*Chronic Hypersensitivity Pneumonitis, a = 39 question instrument, b = 43 question instrument, c = 40 question instrument

After the instrument was drafted, one-on-one semi-structured interviews were conducted in a private room in the WCM Pulmonary outpatient office. This method of in-depth interviewing is widely utilized in qualitative research and allows for a deep dive into the question guide in order to elicit the perspectives of the individual. These one-on-one interviews are commonly used in health care research and have become a standard procedure for conducting cognitive interviews [30, 31]. The interview guide included 11 questions and was written based upon literature review and prior experience of the investigators in writing qualitative interview topic guides [32–34]. (See Supplemental Index A for topic guide). The questions addressed all aspects of the instrument including the instructions, individual item comprehension and suitability, recall period, and response options. An experienced qualitative researcher (KA) conducted all of the interviews. The interviews were audio-recorded and transcribed verbatim and the interviewer took additional field notes. Participants were asked to “think aloud” and talk out their thought processes as they read each question. For each question, participants were asked if there was a suitable response option available. In addition to asking questions of the participants for each item, the interviewer answered “yes” or “no” to three questions for each item: *1. Did the respondent need to re-read or ask you to repeat any part of the question? 2. Did the respondent have any difficulty using the response options? 3. Did the respondent ask for clarification or qualify their answer?* [34] Three rounds of interviews were conducted and the survey was revised in an iterative process (Fig. 1). The transcript data were organized and analyzed using qualitative software (NVivo). Interviews were conducted to saturation in each round (saturation table available in Supplemental Index B and C) [35, 36]. Saturation is the point at which no new information is uncovered about problems with the instructions, items, or response options. The interviews lasted an average of 45 min [37].

### Analytic approach and survey revisions

Data analysis and instrument revisions were completed in an iterative process. Transcripts were analyzed in groups, after each round of interviews was completed. Each transcript was read in detail and the data were then separated and organized by item. Questions where at least two of the respondents identified a problem with comprehension, relevance, clarity, or wording, were edited based upon the feedback given by the respondents. If there was a problem with an item raised by only one participant, a change was made if it represented a significant problem with the survey; otherwise it was discussed amongst the team prior to making the revision. Items were flagged if the interviewer marked “yes” at least once to one of these questions: 1. Did the respondent need to re-read or ask you to repeat any part of the question? 2. Did the respondent have any difficulty using the response options? 3. Did the respondent ask for clarification or qualify their answer? The qualitative comments from the respondents were reviewed for all flagged items and items were revised based upon that feedback. In order to minimize the risk of discrepancy between a patient’s literacy level and their ability to read the survey, readability scores were calculated for the instructions and each individual question using Flesch-Kincaid Reading Ease (FRE) and Flesch-Kincaid Grade Level (F-K) tests [38]. FRE provides a score that corresponds to a grade level. Higher scores correspond to a lower grade level indicating that the content is easier to read. The F-K score reports a grade level that translates to the number of years of education needed to understand the text. Both tests take into account the total number of words, syllables, and sentences in the text being analyzed [39]. Each item was analyzed individually and then the mean, median, range, and standard deviations for all items were calculated for the F-K and FRE. For those items that do not conform to grammatical structure (i.e. those that are 1 or 2 words but share the same question stem) the calculations were performed by including the question stem ahead of each item [40]. Response options were excluded from readability estimates.

## Results

### Participant characteristics

Based upon published guidelines [24] we conducted 10 cognitive interviews. Ten participants were recruited over 3 rounds of interviews, each participant was interviewed once. In round 1, we conducted interviews with the first 5 participants. After revising the instrument we interviewed 3 participants using the revised version and made another round of modifications. To confirm our revised version we conducted interviews with 2 additional participants using the third version. There was a diverse sampling of participants with respect to length of disease, disease severity, and need for supplemental oxygen. Key demographics are highlighted in Table 1.

**Table 1:** Participant characteristics

Variable	Distribution
Median age (range), y	71 (59–85)
Gender	
Female	4 (40%)
Ethnicity	
White	6 (60%)
Hispanic/Latino	2 (20%)
Asian	1 (10%)
Black	1 (10%)
Median number of years living with CHP (range), y	3 (0.17–20)
Education Level	
High School Degree or Equivalent	1 (10%)
Associate Degree	1 (10%)
Bachelor’s degree	4 (40%)
Graduate Degree	4 (40%)
Supplemental Oxygen Use	
Yes	6 (60%)
FVC %	68 (+/−24)
DLCO %	53 (+/−21)
Antigen Identified	
Yes	5 (50%)

Key--*y* years, *CHP* Chronic Hypersensitivity Pneumonitis, *FVC* Forced Vital Capacity, *DLCO* Diffusing Capacity of Carbon Monoxide. FVC and DLCO both presented as mean percent of predicted (+/− standard deviation)

### Instrument revisions

The first version of the instrument included 39 questions. After each round of interviews, revisions to the instrument took place. As the revisions occurred, there was improvement in the number of documented problems by the interviewer (Table 2). The number of items included in the instrument changed twice between the first and fourth versions of the survey. This occurred as items were added and deleted in an iterative process based upon participant feedback. After the first round of interviews (on the 39 item instrument), 4 questions were added based upon participant feedback. For example, a question about environmental triggers of symptoms of CHP such as weather and air pollution was added to distinguish this from environemental triggers that are actual the cause of the disease. After the second round of interviews, three questions that were felt to be redundant or not understandable were deleted. With the combination of the additions and deletions between versions, the final version of the survey that was produced from this content validity phase includes 40 questions.

**Table 2:**
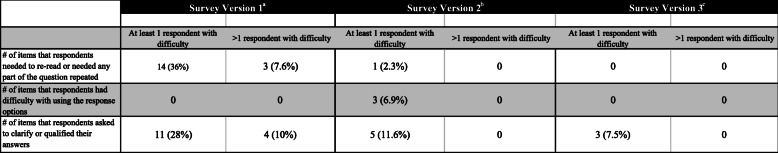
Respondent feedback for items in instrument versions 1–3

Key-- This table indicates the number of items that respondents had difficulty with in each version of the instrument, and what type of difficulty. Columns are split by items in which only one respondent had difficulty vs items where more than one respondent had difficulty

### Assessment of instructions and recall period

All participants reported that they felt comfortable with interpreting the instructions for all parts of the instrument during all three rounds of interviews. In the first round of interviews, we used a two-week recall period. While respondents felt they could easily think back 2 weeks, 4 out of 5 participants (80%) in the first round felt that a four-week recall period was better than a two-week recall period. Respondents explained that 4 weeks provided them with a wider timeframe to reflect upon, as many of the experiences (social events, visits with family, travel) asked about in the instrument may not have occurred in the shorter two-week time period. As such, we modified the recall period from 2 to 4 weeks. Respondents in the second and third rounds agreed that the four-week recall was appropriate.

### Assessment of question structure

During the first round of interviews, there were 7 (18%) questions where more than one participant reported difficulty with comprehension and/or wording (Table 2). In total, 9 (23%) questions were edited after the first round. During the first round, some key words that were misinterpreted were identified. This misinterpretation impacted the responses that several participants provided to questions that included those key words. For example, when the word “fatigue” was written, it was intended to mean tired or lack of energy, separate from shortness of breath. However, two participants out of five interpreted this word to mean the feeling of shortness of breath or breathlessness. Participants commented: *“I don’t know, a heaviness, a trouble catching your breath”.* Similarly, the word “triggers” of your lung condition was intended to mean environmental exposures that are known to cause hypersensitivity pneumonitis. For example, mold and avian exposures are common causes of CHP [41, 42]. .“Triggers” was interpreted differently by four out of five participants in the first round to mean environmental exposures that worsen symptoms but are not necessarily are the cause of the lung disease. Participants commented: “*Yeah, mold is not in my thoughts at all in that question.”, “That’s just fascinating, yea, because I interpreted it a whole different way*”. The questions that contained these terms were re-written to distinguish exposures that are the cause of the lung disease (e.g., mold and avian exposure) from exposures in the environment (e.g., weather or air quality) that may worsen symptoms of the disease. We tracked all revisions made on the items between rounds and examples of these are included in Table 3.

**Table 3:**
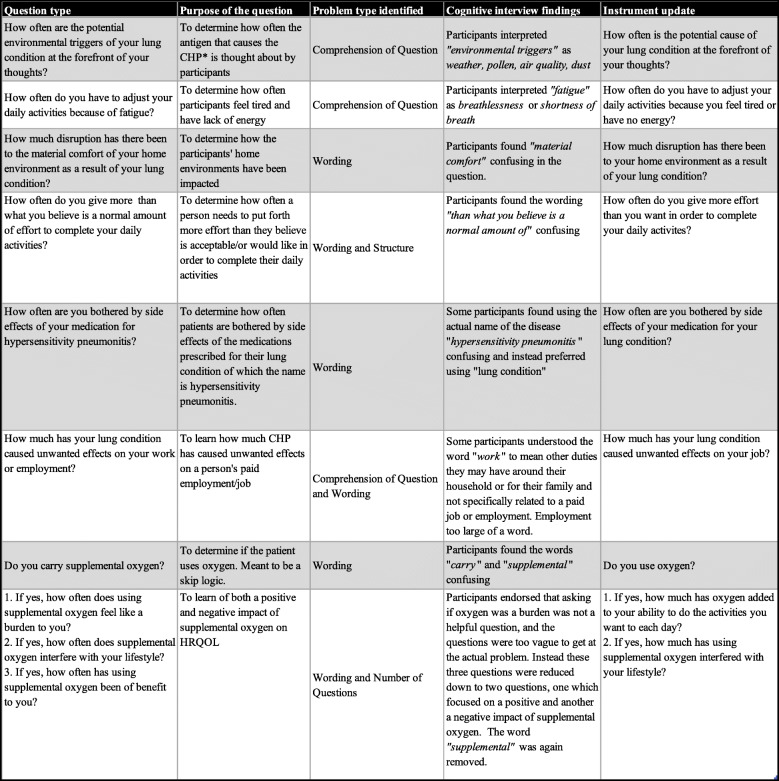
Examples of instrument item revisions using cognitive interview findings from CHP patients

Key-- *Chronic Hypersensitivity Pneumonitis

Some questions had wording with descriptors (e.g. material comfort) that participants found difficult to understand. These descriptors were removed, and wording edited based on suggestions from participants (Table 3). Questions related to use of supplemental oxygen were not relevant for all participants. We added a skip logic, so that the participants were first asked “Do you carry supplemental oxygen?” with instructions to answer the following questions only if they had chosen “yes”. Additional questions that capture both the positive and negative attributes to carrying supplemental oxygen followed this yes/no question. The clarity of these items was evaluated in rounds two and three.

In the second and third rounds of interviews, there were issues reported with question structure and wording (Table 2). By the third round of the interviews, there were no questions where more than one participant found difficulty with comprehension or wording, reflecting improvements that were made after the first and second rounds. Questions related to supplemental oxygen use were modified between the second and third rounds.

### Assessment of response options

The initial instrument utilized 7-point response scales for the first 19 questions, while the remaining 20 questions utilized a 5-point response scale. Two respondents in the first round of interviews noted that 7 response options were too many. For example, when asked “how often” something occurred a respondent commented: “*I’m not sure there is a difference between ‘most of the time’ and ‘a lot of the time’”*, another respondent commented: *“There’s a lot of categories here, that may be too many”*. This scale was reduced to 5 response options and participants thereafter uniformly agreed with a 5-point response scale across all questions. No items were flagged as having issues with response options, their comprehension, or wording.

### Assessment of readability statistics

The questions had a median F-K score of 6.25 (range: 3.8–12.2) and median FRE score of 70.7 (range: 33.6–94.3). The median F-K reflects a 6th–7th grade reading level and falls between “fairly easy” and “standard” in terms of level of difficulty. The instructions had a F-K of 9.5 and FRE score of 65.9. This reflects an 8-9th grade reading level, or “standard” level of difficulty.

## Discussion

Our study used cognitive interviews to refine a CHP-disease specific HRQOL instrument that was developed from a patient-derived quality of life framework [15]. The newly developed CHP-HRQOL instrument addresses a glaring gap in ILD research and practice since no existing HRQOL instrument comprehensively captures the factors that CHP patients have identified as most important [15]. This cognitive interview study outlines the methods of an important step in the validation of the CHP-HRQOL instrument. A disease-specific HRQOL instrument such as this enables healthcare providers to capture domains and symptoms that are most relevant to the CHP patient population, allowing for a more individualized approach to patient assessment. This HRQOL instrument will improve the assessment of the severity of disease and treatment effectiveness in both clinical trials and clinical practice, and clinicians will be equipped to make more individualized treatment recommendations and decisions.

Most of the questions in the first version of the survey were well understood by the participants. Among the items where there was difficulty with comprehension and/or wording, modifications were made based upon participant feedback. By asking participants to read and interpret the questions aloud, we were able to identify difficulties with wording that we may otherwise have missed. We relied on the cognitive interview participants to aid us in crafting questions that are understandable to patients, while at the same time allowing the question to capture the information it is intended to capture. With careful interview transcript analysis and revisions, we identified very few problems with the instrument or individual items by the final round of interviews.

The final version of the survey has a recall period of 4 weeks instead of the two-week timeframe in the initial version of the instrument. As we described in the instrument development phase, there was much discussion amongst the research team about the length of recall period. Initially, 2 weeks was chosen based upon the nature of the disease, the experiences being asked about, and clinical experience in caring for CHP patients. We learned that participants preferred a longer time period, four-weeks, as some of the activities (such as social activities and hobbies that CHP could impact) did not always occur as often as every 2 weeks. There is no single recall period that is best for a HRQOL instrument. When we develop the instrument and choose a recall period, we must take into account the characteristics of phenomena of interest as well as factors that represent the meaning to and experience of the patient [43]. We recognize there may be some weaknesses to longer recall periods including a higher change of variability of responses. While more recently preference has been given to choosing shorter recall periods [29], we relied on our clinical expertise treating CHP patients and patient input to guide the final decision for the four-week recall period. As we are designing a patient-centered instrument, we took the patient input regarding preference for a longer recall period very seriously in this decision. CHP is a chronic illness with minimal day-to-day or week-to week variability in the factors that impact HRQOL, is not a disease that causes cognitive impairment, and clinical visits are often months apart, therefore, we ultimately consider the four-week time frame is appropriate [44]. A longer timeframe (4 weeks or more) is consistent with other commonly utilized and well-validated HRQOL PROs such as the original SF-36, the Saint George’ Respiratory Questionnaire (SGRQ) and the Minnesota Living with Heart Failure Questionnaire (MLHFQ) [45–47]. .Some studies with established PROs have shown equivocal results when comparing shorter and longer recall periods [45]. Longer recall periods are also included in newer PROs that have also taken the patient population and purpose of assessment into context in choosing the recall period such as the Uterine Fibroid Symptom and Health Related Quality of Life questionnaire (UFS-QOL) and the Asthma Impact on Quality of Life Scale (A-QOLS) [48, 49].

After receiving the feedback about the response options from the respondents we ultimately settled on 5 options for all questions. This choice to keep the number of response options is supported by the literature which suggests that dropping unnecessary response options can improve readability of the survey and reduce respondent burden [50]. By eliciting feedback about the response options from the participants we were able to ascertain what options were less likely to be chosen or where there was not much of a discernable difference between two different options, and these were removed to reduce the number of items on the scale.

Although we asked for participants to provide their education level, previous studies have reported that a patient’s educational level is not always consistent with their literacy level [51]. In order to address this, we calculated readability statistics to ensure that the median F-K reading level for the items was at or less than 8th grade, which is the average grade reading level in the United States [52]. We compared this with the estimated scores reported for other HRQOL surveys calculated by F-K and FRE statistics. For example, the SF-36, a well validated generic HRQOL instrument has a median F-K of 4.5 and median FRE level of 79.6 which is lower than the CHP-HRQOL. In contrast, more disease-specific instruments, similar to the CHP-HRQOL, are reported to have higher F-K and FRE scores. For example, the widely used MLHFQ has a median F-K of 9.9 and FRE score of 66.1 [38]. We have also made note of the range of readability estimates and the items with the highest F-K scores in the survey. For example the item “How often do you adjust your daily activities to avoid environmental triggers of your symptoms? (such as weather, air quality, dust)” has an F-K of 12.2, the highest readability score of the survey items. Despite this, we did not discard the item because participants specifically recommended we include this particular item in the survey. In the future validation steps we plan to pay close attention to these particular items for missing item data. In addition we plan to assess comprehension difficulty with particular items in the survey based on education level with a larger diverse cohort of patients.

After revising and refining the instrument we have produced a version that is understandable, appropriately targeted to CHP patients, and measures what it intends to measure. The current stage of the validation process produced a patient-centered survey with 40 items. This instrument is the final version for this stage of validation, however, this is not the final iteration and it is expected that the instrument may change during the next stages of validation as we learn more about the validity and reliability of this individual items. In the next stage of validation, we will administer the instrument to a large and geographically diverse cohort of CHP patients in order to assess the quantitative psychometric properties of the overall instrument and the individual items’ validity and reliability. This instrument will support various lines of scientific inquiry in the future. In these future studies we plan to include analyses of how the heterogeneity of the disease affect’s patient responses to the survey items (for example: disease severity, age, clinical co-mordibidities, and type of antigen source).

This study had limitations. First, while the sample size of participants is consistent with expert recommendations that suggest 7–10 interviews are sufficient to reach saturation with cognitive interviewing [53], we lacked equal representation of individuals with low education levels and minority race/ethnicity. This was due in part to the demographics of the patient population in our clinic. Secondly, the sample of participants was from a single site (referral center), which may limit generalizability. The severity of disease in our patient sample (based on the pulmonary function test results provided in Table 1) is similar to other reported cohorts of CHP patients [14, 54, 55]. To address this further, we plan to administer the survey in a more geographically diverse cohort of patients in the next phase of validation.

## Conclusion

This study is the first to describe, in detail, the documentation of content validity for a disease-specific HRQOL instrument for CHP patients. Through in-depth interviews with CHP patients, we gained valuable feedback about the novel instrument, which allowed us to refine the instrument to make it more appropriate for the target patient population. The CHP-HRQOL instrument is now ready for the next set of validation tests in a larger cohort of patients with CHP. Additionally, this detailed description of the cognitive interview process in a patient population with a less prevalent chronic disease such as CHP can serve as a roadmap for others interested in establishing content validity for a disease-specific PRO.

## Supplementary Information

**Additional file 1.**

**Additional file 2.**

## Data Availability

Data are available from the corresponding author upon reasonable request.

## Abbreviations

A-QOLS: Asthma Impact on Quality of Life Scale
CHP: Chronic Hypersensitivity Pneumonitis
FDA: Food and Drug Administration
F-K: Flesch-Kincaid Grade Level
FRE: Flesch-Kincaid Reading Ease
HRQOL: Health-Related Quality of Life
ILD: Interstitial lung disease
ISPOR: International Society for Pharmacoeconomics and Outcomes Research
MLHFQ: Minnesota Living with Heart Failure Questionnaire
PRO: Patient reported outcome
SGRQ: Saint George’s respiratory questionnaire
SF-36: Short form 36
UFS-QOL: Uterine Fibroid Symptom and Health Related Quality of Life questionnaire
WCM: Weill Cornell Medicine
