# Supplementary material for: Establishing content-validity of a disease-specific health-related quality of life instrument for patients with chronic hypersensitivity pneumonitis
Source: J Patient Rep Outcomes. 2021 Jan 14;5:9. doi: 10.1186/s41687-020-00282-x (PMC7809073; doi:10.1186/s41687-020-00282-x)
Supplement: Supplementary file 1 — Additional file 1. [file 41687_2020_282_MOESM1_ESM.docx]

**Supplemental Index A**: **Cognitive Interview Guide**

**Introduction:**

Thank you for participating in this interview. Your feedback will help us learn how we can best develop survey questions that evaluate quality of life in Hypersensitivity Pneumonitis. The purpose of this interview it to learn what you think about the questions and to make sure they are easily understandable and relevant. Thinking aloud may be new and unfamiliar to you, but please know there are no wrong answers. As we discussed previously, this interview will be audio-recorded. I want to remind you that your confidentiality will be maintained throughout the entire process. What questions do you have before we begin?

**Pre-Interview Warm-Up Question:**

Try to visualize the place where you live, and think about how many windows there are in that place. As you count the windows, tell me what you are seeing and thinking about.

**Interview Questions*:***

The questionnaire is shown to participants. Participants are asked to read one question at a time. The following questions and potential probes will be utilized for each of the items in the survey.

*Questionnaire Instructions:*

1. Please read the instructions for the questionnaire. Can you tell me in your own words what this instruction is asking you to do?

2. Can you describe any confusion or difficulty you had in understanding these instructions?

3. Are there any words or phrases that you would change to improve the instructions?

*Recall Questions:*

1. What does this timeframe mean to you?

2. What period of time did you think about when you were completing the questions?

*Items:*

1. Please look at the question, as you are reading it tell me out loud any thoughts that come to mind.

2. Do you like this question?

3. Is this question easy to understand?

4. Do the response choices make sense?

5. What response choice did you choose? Is this the best response for you?

6. What other thoughts do you have about the question that you have not shared?

*Potential Probes:*

*What made you say that?*

*Why did you respond that way?*

*What does this mean to you? Or what does that word mean to you?*

*Please tell me what this question was asking in your own words*

*Can you take me through the steps of how you came to that answer?*

*What were you thinking when you first answered the question?*

*Tell me more about that*

*For each item, the interviewer will answer the following 3 questions:*

Did the respondent:

1. Need you to repeat any part of the question? Yes (1) No (2)

2. Have any difficulty using the response options? Yes (1) No (2)

3. Ask for clarification or qualify their answer? Yes (1) No (2)
