# Supplementary material for: Establishing content-validity of a disease-specific health-related quality of life instrument for patients with chronic hypersensitivity pneumonitis
Source: J Patient Rep Outcomes. 2021 Jan 14;5:9. doi: 10.1186/s41687-020-00282-x (PMC7809073; doi:10.1186/s41687-020-00282-x)
Supplement: Supplementary file 2 — Additional file 2. [file 41687_2020_282_MOESM2_ESM.docx]

**Supplemental Index B:** **Saturation Table**

|  | **Survey Version 1^a^** | | **Survey Version 2^b^** | | **Survey Version 3^c^** | |
| --- | --- | --- | --- | --- | --- | --- |
|  | At least 1 respondent with difficulty | >1 respondent with difficulty | At least 1 respondent with difficulty | >1 respondent with difficulty | At least 1 respondent with difficulty | >1 respondent with difficulty |
| Total # of issues identified by respondents^d^ | 25 | 7 | 9 | 0 | 3 | 0 |
| # of base issues identified by respondents^e^ |  |  | 25 | 7 | 25 | 7 |
| % change over base (saturation) |  |  | 36% | 0% | 12% | 0% |

Key-- a = 39 questions, 5 participants interviewed; b = 43 questions, 3 participants interviewed; c = 40 questions, 2 participants interviewed; d = the number of items identified by respondents to have issues as indicated by either the number of items respondents needed reread or repeated, difficulty with response options, or needed clarification or to qualify their answer, e = the number of issues respondents identified with survey version 1, which serves as the denominator for the saturation calculations for the subsequent rounds.

In rounds 2 and 3 of the interviews there were no items where more than 1 participant identified the same issue or had difficulty with one particular item.

Reference: (35)

**Supplemental Index C: Mean, median, standard deviation, and range if item readability scores**

| **Calculation** | **Mean** | **SD** | **Median** | **Range** |
| --- | --- | --- | --- | --- |
| F-K^1^ | 6.89 | 2.35 | 6.25 | 3.8-12.2 |
| FRE^2^ | 67.76 | 13.57 | 70.7 | 33.6-94.3 |

Key-- 1 = Flesch-Kincaid Grade Level, 2 = Flesch-Kincaid Reading Ease
